# A heterogeneous label propagation approach to explore the potential associations between miRNA and disease

**DOI:** 10.1186/s12967-018-1722-1

**Published:** 2018-12-11

**Authors:** Xing Chen, De-Hong Zhang, Zhu-Hong You

**Affiliations:** 1 School of Information and Control Engineering, China University of Mining and Technology, Xuzhou, 221116 China; 2 Xinjiang Technical Institute of Physics and Chemistry, Chinese Academy of Science, Ürümqi, 830011 China

**Keywords:** miRNA, Disease, miRNA-disease association, Multi-network, Label propagation

## Abstract

**Background:**

Research on microRNAs (miRNAs) has attracted increasingly worldwide attention over recent years as growing experimental results have made clear that miRNA correlates with masses of critical biological processes and the occurrence, development, and diagnosis of human complex diseases. Nonetheless, the known miRNA-disease associations are still insufficient considering plenty of human miRNAs discovered now. Therefore, there is an urgent need for effective computational model predicting novel miRNA-disease association prediction to save time and money for follow-up biological experiments.

**Methods:**

In this study, considering the insufficiency of the previous computational methods, we proposed the model named heterogeneous label propagation for MiRNA-disease association prediction (HLPMDA), in which a heterogeneous label was propagated on the multi-network of miRNA, disease and long non-coding RNA (lncRNA) to infer the possible miRNA-disease association. The strength of the data about lncRNA–miRNA association and lncRNA-disease association enabled HLPMDA to produce a better prediction.

**Results:**

HLPMDA achieved AUCs of 0.9232, 0.8437 and 0.9218 ± 0.0004 based on global and local leave-one-out cross validation and 5-fold cross validation, respectively. Furthermore, three kinds of case studies were implemented and 47 (esophageal neoplasms), 49 (breast neoplasms) and 46 (lymphoma) of top 50 candidate miRNAs were proved by experiment reports.

**Conclusions:**

All the results adequately showed that HLPMDA is a recommendable miRNA-disease association prediction method. We anticipated that HLPMDA could help the follow-up investigations by biomedical researchers.

**Electronic supplementary material:**

The online version of this article (10.1186/s12967-018-1722-1) contains supplementary material, which is available to authorized users.

## Background

MicroRNAs (miRNAs) consist of about 22 nucleotides and they are one category of endogenous short non-coding RNAs (ncRNAs) that could regulate the expression of target messenger RNAs (mRNAs) at the level of transcription and post-translation [1–4]. There are 28645 miRNAs in the 21st version of miRBase [5] including more than three thousand human miRNAs. As regulators of gene expression and protein production, on the one hand some of miRNAs serve as negative regulators by binding to the 3′-UTRs of the target mRNAs [4]; on the other hand, the regulatory impact of some miRNAs is positive [6, 7]. Thus miRNAs have effect on cell proliferation [8], development [9], differentiation [10], apoptosis [11], metabolism [12, 13], aging [12, 13], signal transduction [14], and viral infection [10]. Moreover, evidence is mounting that miRNAs play a fundamental role in the development, progression, and prognosis of numerous human diseases [15–20]. For instance, HIV-1 replication could be enhanced by miR-132 [21] and similarly, cocaine could down-regulate miR-125b in CD4+ T cells to enhance HIV-1 replication [22]. Breast neoplasms stem cell formation could be promoted by downregulation of miR-140 in basal-like early stage breast cancer [23]. In addition, compared to normal epithelium, miR-139 and miR-140 was down-regulated during lobular neoplasia progression [24]. The transcripts of certain let-7 homologs would be downregulated in human lung cancer and the low levels of let-7 would link to poor prognosis [25]. In addition, non-small-cell lung cancer relates to many other miRNAs [26–29].

Faced with a great variety of miRNAs and diseases, experimental methods for the sake of finding new associations between miRNAs and diseases, are both costly and time-consuming. In the wake of the growth of the biological datasets, the practicable computational methods are urgently necessary to greatly help identify more disease-related miRNAs and explore new perspective treatment of various important human diseases. Over the past decade, some progress has been made to uncover novel miRNA-disease associations. Most computational methods depends on the assumption that functionally similar miRNAs usually have connection with phenotypically similar diseases [30–36]. From the standpoints of network and systems biology, most computational methods belonged to the similarity measure-based approaches or machine learning-based approaches.

A functionally related miRNA network and a human phenome-microRNAome network were first constructed by Jiang et al. [37]. Then the disease phenotype similarity network, miRNA functional similarity network, and the known human disease-miRNA association network were combined together. Based on the combination, they devised a computational model of disease-miRNA prioritization, which could rank the entire human microRNAome for investigated diseases. However, its prediction performance was ordinary because of only using miRNA neighbor information. Furthermore, Xuan et al. [38] proposed HDMP model to predict disease-related miRNA candidates on the basis of weighted *k* most similar neighbors. In HMDP, miRNA functional similarity was calculated through the information content of disease terms and disease phenotype similarity. Then, the miRNA family (cluster) information was considered and miRNA functional similarity was recalculated after giving higher weight to members in the same miRNA family (cluster). However, the precision was directly influenced by the number of a miRNA’s neighbors. These two methods were limited by their local network similarity measure, which meant it was insufficient to simply consider miRNA neighbor information. Therefore, global network similarity measure was adopted in some studies. Chen et al. [39] proposed Random Walk with Restart for MiRNA-disease association (RWRMDA), in which random walk analysis was applied to miRNA–miRNA functional similarity network. It was a pity that this method was the unavailability for diseases with no confirmed related miRNAs despite of its passable predictive accuracy. Xuan et al. [40] further put forward a random walk method, MIDP, in which transition weights of labeled nodes were higher than unlabeled nodes. In MIDP, the side effect of the noisy data was reduced by fitting restart rate and MIDP is applicable for the disease with no related miRNAs.

Some other methods made use of the information about confirmed disease-related genes and predicted miRNA-target interactions. For instance, Shi et al. [41] developed a computational prediction method in which random walk analysis was used in the protein–protein interaction (PPI) networks. It is assumed that if a target gene of a miRNA associates with a disease, this disease is likely to be related with the miRNA. MiRNA-target interactions and disease-gene associations were integrated into a PPI network and then the functional relationship information about miRNA targets and disease genes was dug out in this PPI network. Besides, this method could serve to find miRNA-disease co-regulated modules by hierarchical clustering analysis. Mørk et al. [42] presented miRPD in which miRNA-protein-disease associations, not just miRNA-disease associations, were predicted. It was a good idea to bring in the abundant information of protein as a bridge indirectly linking the miRNA and the disease. In detail, known and predicted miRNA-protein associations were coupled with protein-disease associations from the literature to make an inference about miRNA-disease associations. In fact, the molecular bases for human diseases we had partly known accounted for less than 40% and highly accurate miRNA-target interactions can hardly be obtained. In other words, above two methods lacked solid data foundation. Chen et al. [43] proposed a model based on super-disease and miRNA for potential miRNA-disease association prediction (SDMMDA). In view of the fact that rare miRNA-disease associations were known and many associations are ‘missing’, the concepts of ‘super-miRNA’ and ‘super-disease’ were introduced to improve the similarity measures of miRNAs and diseases.

The computational methods based on machine learning could bring us some new inspiration. Xu et al. [44] constructed the miRNA-target dysregulated network (MTDN) and introduced support vector machine (SVM) classifier based on the features and changes in miRNA expression to distinguish positive miRNA-disease associations from negative associations. However, there was little confirmed information about negative samples, so improvement was needed. In view of the lack of negative samples, Chen et al. [45] developed a semi-supervised method named Regularized Least Squares for MiRNA-disease association (RLSMDA). In the framework of regularized least squares, RLSMDA was a global method integrating disease semantic similarity, miRNA functional similarity and human miRNA-disease associations. RLSMDA could simultaneously prioritize all the possible miRNA-disease associations without the need of negative samples. Chen et al. [46] proposed Restricted Boltzmann machine for multiple types of miRNA-disease association prediction (RBMMMDA) by which four types of miRNA-disease associations could be identified. RBMMMDA is the first model which could identify different types of miRNA-disease associations. There is a hypothesis that by distributional semantics, information attached to miRNAs and diseases can be revealed. Pasquier and Gardès [47] developed a model named MirAI, in which the hypothesis was investigated by expressing distributional information of miRNAs and diseases in a high-dimensional vector space and then associations between miRNAs and diseases could be defined considering their vector similarity. Chen et al. [39] introduced KNN algorithm into miRNA-disease association prediction and proposed the computational model of RKNNMDA (Ranking-based KNN for MiRNA-disease association prediction).

Some previous researches paid attention to the network tool-based prediction model. For instance, Xuan et al. [40] divided network nodes into labeled nodes and unlabeled nodes and gave them different transition weights. The restart of walking could determine the walking distance, so the negative effect of noisy data would be lessened. Specially, the information from different layers of the miRNA-disease bilayer network was weighed differently. Then, Chen et al. [48] developed Within and Between Score for MiRNA-disease association prediction (WBSMDA) in which for the first time, Gaussian interaction profile kernel similarity for diseases and miRNAs were combined with miRNA functional similarity, disease semantic similarity and miRNA-disease associations. Chen et al. [49] further proposed Heterogeneous graph inference for miRNA-disease association prediction (HGIMDA) and the heterogeneous graph was constructed by the combination of miRNA functional similarity, disease semantic similarity, Gaussian interaction profile kernel similarity, and miRNA-disease associations. Similar to random walk, HGIMDA was an iterative process for the optimal solutions based on global network similarity. In aspect of AUC, HGIMDA reached 0.8781 and 0.8077 after implementing global and local LOOCV, respectively. Li et al. [50] put forward MCMDA (Matrix Completion for MiRNA-disease association prediction) in which a matrix completion algorithm was introduced and the lowly ranked miRNA-disease matrix was updated efficiently. WBSMDA, HGIMDA and MCMDA apply to the disease (miRNA) without any proved related miRNAs (diseases). MaxFlow is a combinatorial prioritization algorithm proposed by Yu et al. [51]. Besides the same type of data used in WBSMDA, MaxFlow also introduced the information about disease phenotypic similarity, miRNA family and miRNA cluster. Then a directed miRNAome-phenome network graph was constructed and every weighted edges were seen as flow capacity. The association possibility was defined as the flow quantity from the miRNA node to the investigated disease node. You et al. [52] proposed Path-Based computational model for MiRNA-disease association prediction (PBMDA). A heterogeneous graph, including three interlinked sub-graphs, was constructed by the same data as in WBSMDA and depth-first search algorithm was applied to predict possible existing miRNA-disease associations. Chen et al. [53] summed up the relatively important miRNA-disease association prediction approach.

More links should exist between miRNAs and diseases than we had learned. However, the computational methods aforementioned were limited by the utilization of inaccurate information (such as miRNA-target interactions), the selection of parameter values, the combination of different classifiers in the different networks or spaces, etc. In pursuit of the higher predictive accuracy, we proposed heterogeneous label propagation for MiRNA-disease association prediction (HLPMDA) for underlying miRNA-disease association prediction. In HLPMDA, heterogeneous data (miRNA similarity, disease similarity, miRNA-disease association, long non-coding RNA (lncRNA)-disease association and miRNA–lncRNA interaction) were integrated into a heterogeneous network [54]. Then, disease-related miRNA prioritization problem was formulated as an optimization problem. In details, within-network smoothness and cross-network consistency were considered here. HLPMDA achieved AUCs of 0.9232, 0.8437 and 0.9218 ± 0.0004 based on global/local LOOCV and 5-fold cross validation, respectively. Both in local and global LOOCV, HLPMDA was better than previous methods. In the case studies of three human diseases, 47, 49 and 46 out top 50 predicted miRNAs for esophageal neoplasms, breast neoplasms and lymphoma were verified by some recent experimental research.

## Methods

### Human miRNA-disease associations

There are 5430 human miRNA-diseases associations between 383 diseases and 495 miRNAs, which were obtained from the Human microRNA Disease Database version 2.0 [55]. For convenience, the adjacency matrix *S*_1,2_ represented known miRNAs-disease associations. If miRNA *m(j)* is associated with disease *d(i)*, *S*_1,2_*(i, j)* = 1; otherwise, *S*_1,2_*(i, j)* = 0. In addition, variable *nm* and *nd* indicated the number of involved miRNAs and diseases, respectively.

### lncRNA-disease associations

Because we aim to predict latent miRNA-disease association, we looked for the lncRNAs that associate with the disease contained in *S*_1,2_, or interacted with the miRNAs contained in *S*_1,2_. As a result, 1089 lncRNAs (from LncRNADisease database [56] and starBase v2.0 database [57] matched the above conditions. For the convenience of subsequent calculations, the adjacency matrix $S_{2,3} \in R^{383 \times 1089}$ was constructed to represent known lncRNA-disease associations. If lncRNA *l(j)* is associated with disease *d(i)*, *S*_2,3_ *(i, j)* = 1; otherwise, *S*_2,3_ *(i, j)* = 0. Variable *nl* means the number of involved lncRNAs. The known lncRNA-disease associations came from LncRNA disease database (http://www.cuilab.cn/lncrnadisease) which provided many experimentally confirmed lncRNA-disease associations and we deleted duplicate associations with different evidences. Finally 251 different confirmed lncRNA-disease associations were selected out and in fact they only had something to do with 150 lncRNAs and 63 diseases so *S*_2,3_ was a sparse matrix.

### miRNA–lncRNA interactions

Similarly, the adjacency matrix $S_{1,3} \in R^{495 \times 1089}$ was constructed to represent known miRNA–lncRNA interaction. If miRNA *ms(i)* is interacted with lncRNA *l(j)*, *S*_1,3_ *(i, j)* = 1; otherwise, *S*_1,3_ *(i, j)* = 0. MiRNA–lncRNA interaction dataset was downloaded from starBase v2.0 database [57] (http://starbase.sysu.edu.cn/), which provided the most comprehensive experimentally confirmed miRNA–lncRNA interactions based on large scale CLIP-Seq data. Then we deleted duplicate interactions and 9088 different confirmed lncRNA–miRNA interactions were selected out. Similar to *S*_2,3_, *S*_1,3_ was also a sparse matrix in which the interactions were only about 246 miRNAs rather than all the 495 miRNAs.

### MiRNA functional similarity

It was assumed in the previous work [58] that functional similar miRNAs often correlate with phenotypically similar diseases. Based on this important assumption, miRNA functional similarity score was calculated and the related data could be downloaded from http://www.cuilab.cn/files/images/cuilab/misim.zip. Analogously, the miRNA functional similarity network was represented by miRNA functional similarity matrix *FS*, in which functionally similar between miRNA *m(i)* and *m(j)* is denoted by the entity *FS(m(i), m(j))*.

### Disease semantic similarity model

**Fig. 1:**
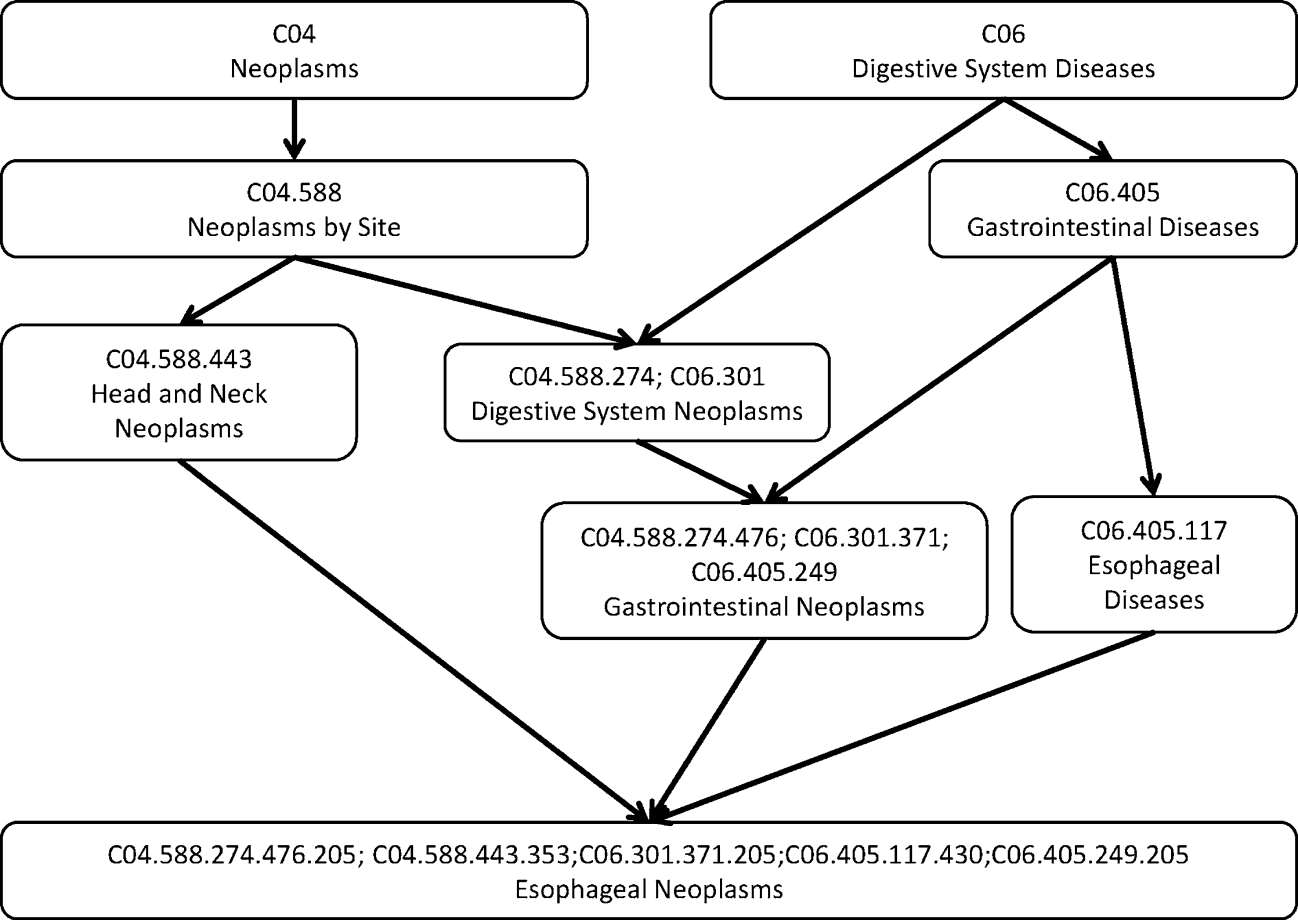
The disease DAG of esophageal neoplasms

There are two kinds of models to calculate disease semantic similarity. Directed acyclic graph (DAG) is a finite directed graph but there is no directed circle in it. DAG consists of finite vertices and edges, with each edge directed from one node (parent) to another (child), and it is impossible to start at a node *n* and follow a consistently-directed sequence of edges that eventually loops back to *n* again. DAG served as a tool to describe the relationships among involved diseases in many previous studies [45, 48, 49, 52]. According to the data from the National Library of Medicine (http://www.nlm.nih.gov/), the relationship of different diseases could be measured by the disease DAG based on the MeSH descriptor of Category C. For example, for the DAG of esophageal neoplasms (see Fig. 1), ‘Neoplasms’ points to ‘Neoplasms by Site’, so ‘Neoplasms’ is the parent of child ‘Neoplasms by Site’. The disease *D* was represented by *DAG(D)* = *(D,T(D),E(D))*, in which *T(D)* is the node set representing disease *D* itself and its ancestor (its parent and above), *E(D)* is the corresponding direct edges from the parent to the child [58]. According to [38], the semantic value of disease *D* could be calculated as follows:

1 $$\begin{array}{*{20}c} {DV\left( D \right) = \mathop \sum \limits_{d \in T\left( D \right)} D_{D} \left( d \right)} \\ \end{array}$$

where

2 $$\begin{array}{*{20}l} {D_{D} \left( d \right) = \left\{ {\begin{array}{*{20}c} {1, } & \quad {if \;d = D} \\ {\text{max} \left\{ {\Delta *D_{D} \left( {d^{\prime}} \right) |d^{\prime} \in children \;of\;d} \right\},} & \quad {if\; d \ne D} \\ \end{array} } \right.} \\ \end{array}$$

where ∆ is the semantic contribution factor. For disease *D*, the contribution of itself to the semantic value of disease *D* was 1 and the longer distance between *D* and other disease was, the smaller semantic contribution was. If disease terms are in the same layer, they would have the same contribution to the semantic value of disease *D*.

There is a wildly accepted assumption that the more part of two diseases’ DAGs are sharing, the more semantic similarity they have. The semantic similarity between disease *d(i)* and *d(j)* can be defined as follows:

3 $$\begin{array}{*{20}c} {DS1\left( {d\left( i \right),d\left( j \right)} \right) = \frac{{\mathop \sum \nolimits_{{t \in T\left( {d\left( i \right)} \right) \cap T\left( {d\left( j \right)} \right)}} D_{d\left( i \right)} \left( t \right) + D_{d\left( j \right)} \left( t \right)}}{{DV\left( {d\left( i \right)} \right) + DV\left( {d\left( j \right)} \right)}}} \\ \end{array}$$

Furthermore, there is another model for disease similarity calculation [38] and it was adopted in this study. It is observed that in the same layer of *DAG(A)*, different diseases terms may appear in the different numbers of disease DAGs. For instance, there are two diseases in the same layer of *DAG(A)*, if one disease appears in less disease DAGs than the other, it is obvious that the former is more specific than the latter. So we assigned them with different contributions, and the former’s contribution factor should be higher than the latter. The contribution of disease term *t* in *DAG(A)* to the semantic value of disease *A* is defined as follows:

4 $$\begin{array}{*{20}c} {C2A\left( t \right) = - \log \left( {\frac{{DAG_{t} }}{nd}} \right)} \\ \end{array}$$

where DAG_t_ represents the number of DAGs including *t*. The semantic similarity between two diseases were defined as follows:

5 $$\begin{array}{*{20}c} {DS2\left( {d\left( i \right),d\left( j \right)} \right) = \frac{{\mathop \sum \nolimits_{t \in T\left( A \right) \cap T\left( B \right)} C2_{A} \left( t \right) + C2_{B} \left( t \right)}}{{C2\left( {d\left( i \right)} \right) + C2\left( {d\left( j \right)} \right)}}} \\ \end{array}$$

So the final disease semantic similarity was defined as follows:

6 $$\begin{array}{*{20}c} {DS = \frac{DS1 + DS2}{2}} \\ \end{array}$$

### Gaussian interaction profile kernel similarity for diseases and miRNAs

In order to make the most of the topologic information from known miRNA-disease association network, Gaussian interaction profile kernel similarity for diseases are calculated on the assumption that analogic diseases are likely to associate with functionally similar miRNAs and vice versa [20, 58–60]. The *i*th row of the adjacency matrix *S*_1,2_ is taken out as a new binary vector, *IP(d(i))*. Obviously, *IP(d(i))* illustrate the associative or non-associative situation between disease *d(i)* and all miRNAs involved in this study and it is called interaction profiles of disease *d(i)*. According to [61], Gaussian kernel similarity between two diseases, *d(i)* and *d(j)*, could be calculated as follows:

7 $$\begin{array}{*{20}c} {KD\left( {d\left( i \right), d\left( j \right)} \right) = exp\left( { - \gamma_{d} \left\| {IP\left( {d\left( i \right)} \right) - IP\left( {d\left( j \right)} \right)} \right\|^{2} } \right)} \\ \end{array}$$

where *γ*_*d*_ is a parameter for the kernel bandwidth control, and it was calculated through the normalization of a new bandwidth parameter $Y^{\prime}_{d}$ by the average number of associations with miRNAs for all the diseases.

8 $$\begin{array}{*{20}c} {\gamma_{d} = \frac{{\gamma^{\prime}_{d} }}{{\frac{1}{nd}\mathop \sum \nolimits_{i = 1}^{nd} \left\| {IP\left( {d\left( i \right)} \right)} \right\|^{2} }}} \\ \end{array}$$

Similarly, Gaussian interaction profile kernel similarity between two miRNAs (*m(i)* and *m(j)*) is calculated as follows:

9 $$\begin{array}{*{20}c} {KM\left( {m\left( i \right),m\left( j \right)} \right) = exp\left( { - \gamma_{m} \left\| {IP\left( {m\left( i \right)} \right) - IP\left( {m\left( j \right)} \right)} \right\|^{2} } \right)} \\ \end{array}$$

10 $$\begin{array}{*{20}c} {\gamma_{m} = \gamma^{\prime}_{m} /\left( {\frac{1}{nm}\mathop \sum \limits_{i = 1}^{nm} \left\| {IP\left( {m\left( i \right)} \right)} \right\|^{2} } \right)} \\ \end{array}$$

where $IP\left( {m\left( i \right)} \right) \;{\text{and}}\; IP\left( {m\left( j \right)} \right)$ represent *i*th column and the *j*th column of the adjacency matrix *S*_1,2_; *γ*_*m*_ is a parameter for the kernel bandwidth control, and it was calculated through the normalization of a new bandwidth parameter $Y^{\prime}_{m}$ by the average number of associated diseases for all the miRNAs. According to [62] and for the simplicity of calculations, we set *γ*_*d*_ = *γ*_*m*_ = 1.

### Integrated similarity for miRNAs and diseases

Here, according to [48], let *S*_1_ represent the integrated miRNA similarity matrix and *S*_2_ be the integrated disease similarity matrix.

11 $$S_{1} \left( {m\left( i \right),m\left( j \right)} \right) = \left\{ {\begin{array}{*{20}l} {FS\left( {m\left( i \right),m\left( j \right)} \right), } & \quad {if\;m\left( i \right)\;{\text{and}}\;m\left( j \right)\;{\text{have}}\;{\text{functional}}\;{\text{similarity}}} \\ {KM\left( {m\left( i \right),m\left( j \right)} \right), } & \quad { {\text{otherwise}}} \\ \end{array} } \right.$$

12 $$S_{2} \left( {d\left( i \right),d\left( j \right)} \right) = \left\{ {\begin{array}{*{20}l} {DS\left( {d\left( i \right),d\left( j \right)} \right),} & \quad {if\;d\left( i \right)\;{\text{and}}\;d\left( j \right) \;{\text{have}}\;{\text{semantic}}\;{\text{similarity}}} \\ {KD\left( {d\left( i \right),d\left( j \right)} \right),} & \quad {\text{otherwise}} \\ \end{array} } \right.$$

### HLPMDA

**Fig. 2:**
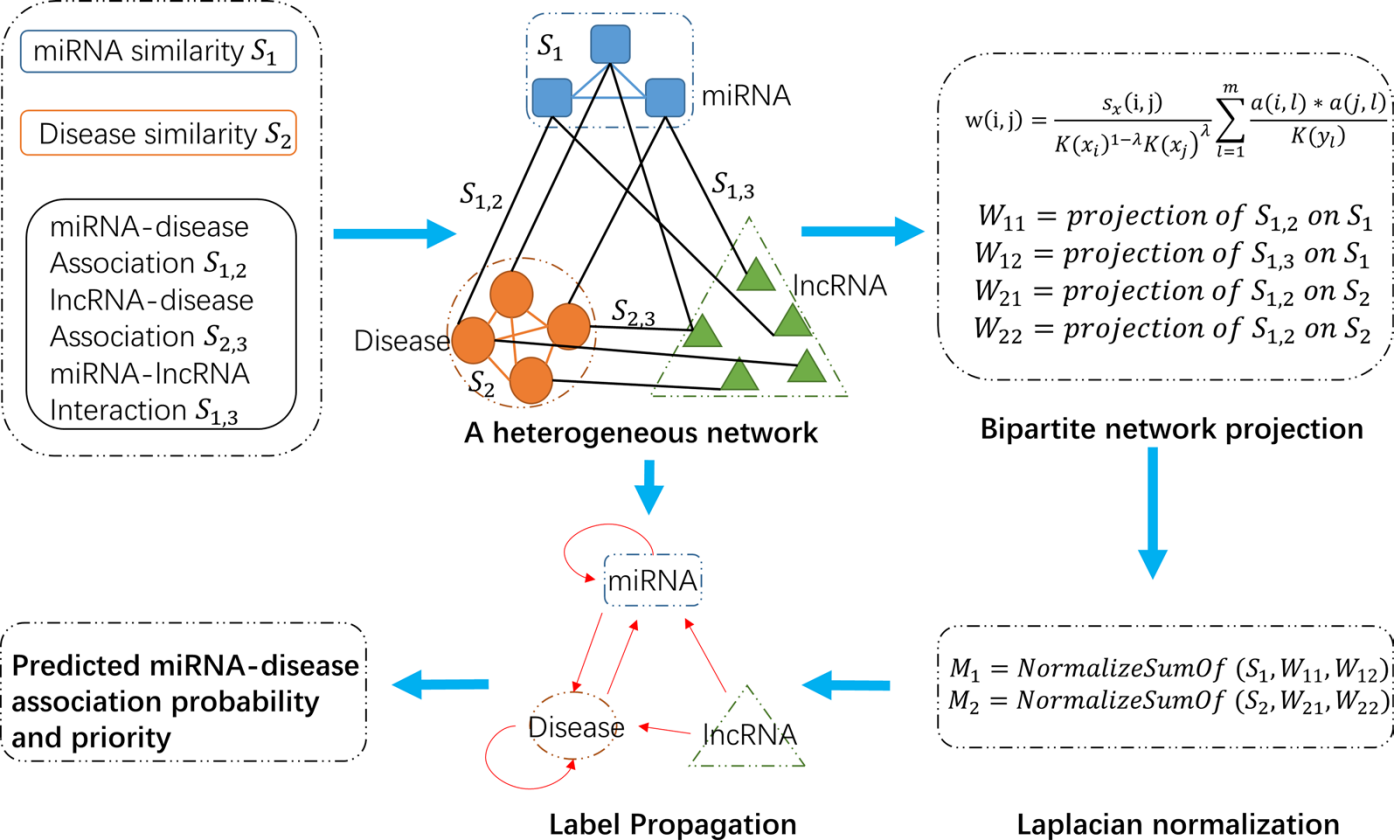
Flowchart of possible disease-miRNA association prediction based on the computational model of HLPMDA

HLPMDA is motivated by Heter-LP [63]. As shown in Fig. 2, the heterogeneous network constructed based on the above data included three kinds of nodes (miRNAs, diseases, and lncRNAs) and five kinds of edges (miRNA similarity, disease similarity, miRNA-disease association, miRNA–lncRNA interaction and lncRNA-disease association). Thus a heterogeneous network *G *=* (V, E)* was constructed with two homo-sub-networks and three hetero-sub-networks (see Fig. 2). The homo-sub-networks are defined as *G*_*i*_=* (V*_*i*_*,E*_*i*_*)* where *i* = 1, 2 for miRNAs and diseases, respectively. The hetero-sub-networks (bipartite networks) are $G_{i,j} = (V_{i} \cup V_{j} , \, E_{i,j} )\;{\text{for}}\;i, \, j = { 1},{ 2},{ 3},\;{\text{and}}\;i \, < \, j,$ where *i,j* = 1, 2, 3 for miRNAs, diseases and lncRNAs, respectively. *E*_*i*_ represents the set of edges between vertices in the vertex set *V*_*i*_ of homo-sub-network *G*_*i*_. And *E*_*i,j*_ represents the set of edges between a vertex in *V*_*i*_ to a vertex in *V*_*j*_.

On the base of heterogeneous network *G*, we measure the weight of homo-sub-network edge (*i, j*) by bipartite network projection, a weighted one-mode projection technique from [63, 64]. Let the adjacency matrix *A* represent one bipartite network, in which there are two nonempty disjoint vertex sets *X* and *Y*. *S*_*x*_ is the similarity matrix of vertex set *X* and *s*_*x*_ *(i, j)* is the entry of row *i* and column *j* in *S*_*x*_; *K*(*x*_*i*_) represents the degrees of vertices *x*_*i*_ in *G*; *W* is the projected matrix of *A* onto *X* and the corresponding calculation process is:

13 $$\begin{array}{*{20}c} {w\left( {i,j} \right) = \frac{{s_{x} \left( {i,j} \right)}}{{K\left( {x_{i} } \right)^{1 - \lambda } K\left( {x_{j} } \right)^{\lambda } }}\mathop \sum \limits_{l = 1}^{m} \frac{{a\left( {i,l} \right)*a\left( {j,l} \right)}}{{K\left( {y_{l} } \right)}}} \\ \end{array}$$

where *i,j* belong to identical homo-sub-networks; *w(i, j)* is the entry of row *i* and column *j* in *W*; 0 <* k* < 1 is diffusion parameter of the projection (in this study we set *k* = 0.5); *a*(*i*, *l*) represents the weight of edge (*x*_*i*_, *y*_*l*_) in *G*. If there is no edge from *i* to *j*, *w*(*i*, *j*) = 0.

Next, label propagation was applied on miRNA-disease hetero-sub-network by means of the information from other homo-sub-networks and hetero-sub-networks. Table 1 shows the main pseudo-code of HLPMDA. Firstly, let *y*_1_, *y*_2_ and *y*_3_ be the label vectors that represent miRNA, disease and lncRNA, respectively. *y*_1_, *y*_2_ and *y*_3_ were initialized to zero. Secondly, all associations (*S*_1,2_ and *S*_2,3_) and interactions (*S*_1,3_) were projected onto similarity matrices (*S*_1_ and *S*_2_) using the weighted one-mode projection technique as described above. Four projected matrices came out (*W*_11_ is the projection of *S*_1,2_ on *S*_1_; *W*_12_ is the projection of *S*_1,3_ on *S*_1_; *W*_21_ is the projection of *S*_1,2_ on *S*_2_; *W*_22_ is the projection of *S*_2,3_ on *S*_2_). Thirdly, four projected matrices ($W_{11} , W_{12}$ and $W_{21} , W_{22}$) were integrated with corresponding similarity matrices (*S*_1_ or *S*_2_) respectively, with the help of the Laplacian normalization (*M*_1_ is the Laplacian normalization of $S_{1} , W_{11}$ and $W_{12}$; *M*_2_ is the Laplacian normalization of $S_{2} , W_{21}$ and *W*_22_). Taking *M*_1_ as an example, the Laplacian normalization is defined by

14 $$\begin{array}{*{20}c} {M\left( {{\text{i}},{\text{j}}} \right) = S_{1} \left( {{\text{i}},{\text{j}}} \right) + W_{11} \left( {{\text{i}},{\text{j}}} \right) + W_{12} \left( {{\text{i}},{\text{j}}} \right)} \\ \end{array}$$

15 $$\begin{array}{*{20}c} {M\left( {{\text{i}},{\text{j}}} \right) = \left\{ {\begin{array}{*{20}c} {1,} & {i = j} \\ {\frac{{M\left( {{\text{i}},{\text{j}}} \right)}}{{\sqrt {d\left( i \right)d\left( j \right)} }},} & {i \ne j} \\ \end{array} } \right.} \\ \end{array}$$

where *d*(*i*) is the sum of *i*th row of the matrix *M*, and if *d*(*i*) = 0, *d*(*i*) = 1.

**Table 1:**
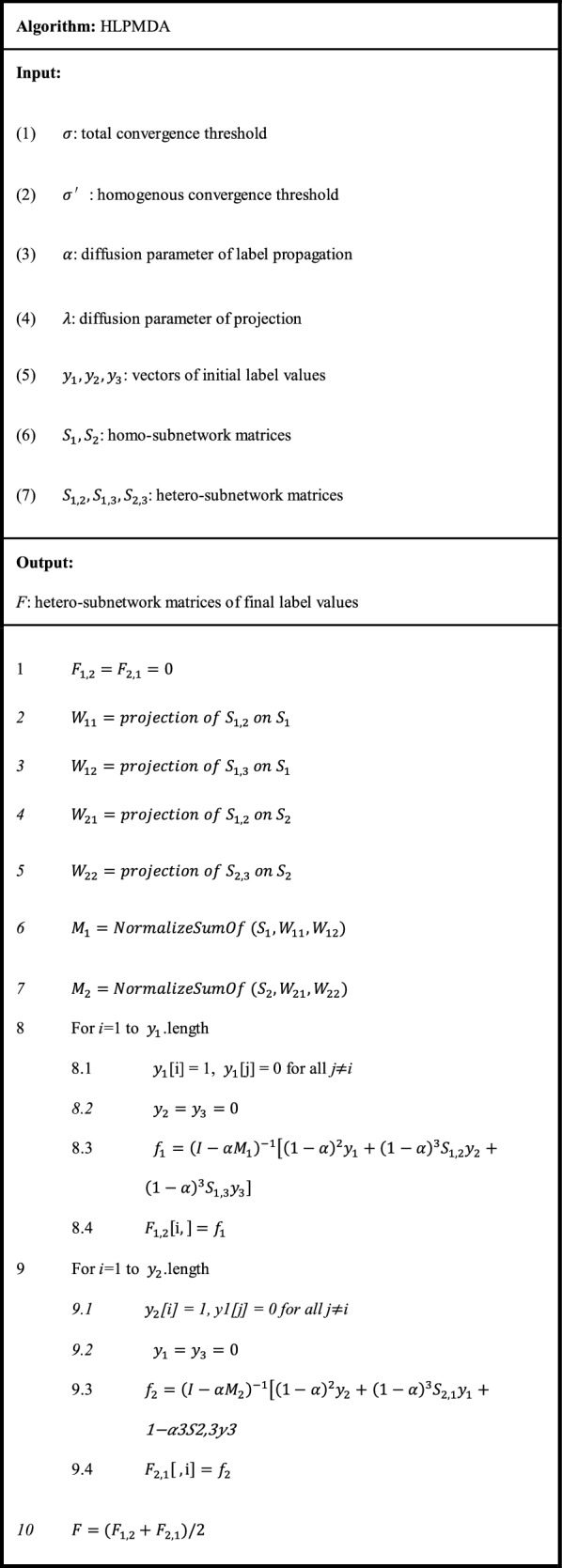

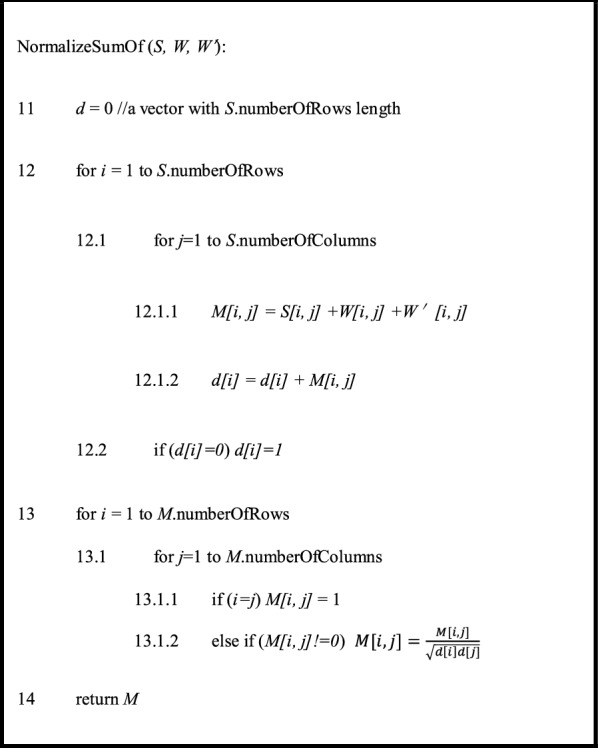
The illustration of the HLPMDA algorithm

Then in label propagation phase, there were three iterative loops. In each loop, the label of the investigated miRNA (disease or lncRNA) was set to one and others to zero. The label propagation function is applied, and output matrices, *F*_1,2_ and *F*_2,1_, are updated. Finally, the predictive matrix *F* for underlying miRNA-disease associations could be obtained and then all predictive scores could be ranked in descending order.

According to the previous study [63], the convergence of label propagation iteration (*LabelPropagation* function) in the algorithm HLPMDA could be determined (the relevant proof can be found in [63]). So in order to reduce the time complexity and space complexity of HLPMDA, the complex part, i.e. *LabelPropagation* function was replaced by the following equation:

16 $$\begin{array}{*{20}c} {f_{1} = \left( {I - \alpha M_{1} } \right)^{ - 1} \left[ {\left( {1 - \alpha } \right)^{2} y_{1} + \left( {1 - \alpha } \right)^{3} S_{1,2} y_{2} + \left( {1 - \alpha } \right)^{3} S_{1,3} y_{3} } \right]} \\ \end{array}$$

17 $$\begin{array}{*{20}c} {f_{2} = \left( {I - \alpha M_{2} } \right)^{ - 1} \left[ {\left( {1 - \alpha } \right)^{2} y_{2} + \left( {1 - \alpha } \right)^{3} S_{2,1} y_{1} + \left( {1 - \alpha } \right)^{3} S_{2,3} y_{3} } \right]} \\ \end{array}$$

where *f*_1_ and *f*_2_ are label vectors that represent the predictive result for the investigated miRNA with all diseases or the investigated disease with all miRNAs; *I* is the identity matrix;$S_{2,1} = \left( {S_{2,1} } \right)^{T}$; *α* is a constant parameter and we set *α* = 0.1 referring to the similar study [63].

## Results

### Cross validation

In order to evaluate the predictive performance of HLPMDA, global LOOCV, local LOOCV and 5-fold cross validation were executed based on the known miRNA-disease associations from HMDD v2.0 [55]. Then, HLPMDA was compared with ten state-of-the-art computational methods: PBMDA [52], MCMDA [50], MaxFlow [51], HGIMDA [49], RLSMDA [45], HDMP [38] WBSMDA [48], MirAI [47], MIDP [40] and RWRMDA [65].

In LOOCV, each proved miRNA-disease association was regarded as a test sample in turn while other known associations were used as training set of the model. The difference between local and global LOOCV is the comparison range. In local LOOCV, a comparison was made between test sample and the miRNAs without known association with the investigated disease. Whereas in global LOOCV, a comparison was made between test sample and all the miRNA-disease pairs without confirmed associations. In 5-fold cross validation, all the known miRNA-disease associations in HMDD v2.0 were divided into five sets with equal sizes, where four sets trained the model and the other set tested the model. For fear of the performance difference due to the samples divisions, all associations were randomly divided 100 times and the results of all 100 times were averaged to derive the final evaluation result.

If the test sample ranked higher than the given threshold, it was a successful prediction. Next, Receiver operating characteristics (ROC) curve was drawn where true positive rate (TPR, sensitivity) was plotted versus false positive rate (FPR, 1-specificity) at different thresholds. Sensitivity represents the ratio of successful predictions to the test samples. Specificity represents the percentage of negative miRNA-disease pairs which were ranked lower than the threshold. Area under the ROC curve (AUC) could be calculated to show predictive capability of MDMMDA. The closer that AUC is to 1, the better predictive capability the method is. AUC = 0.5 means the random performance.

**Fig. 3:**
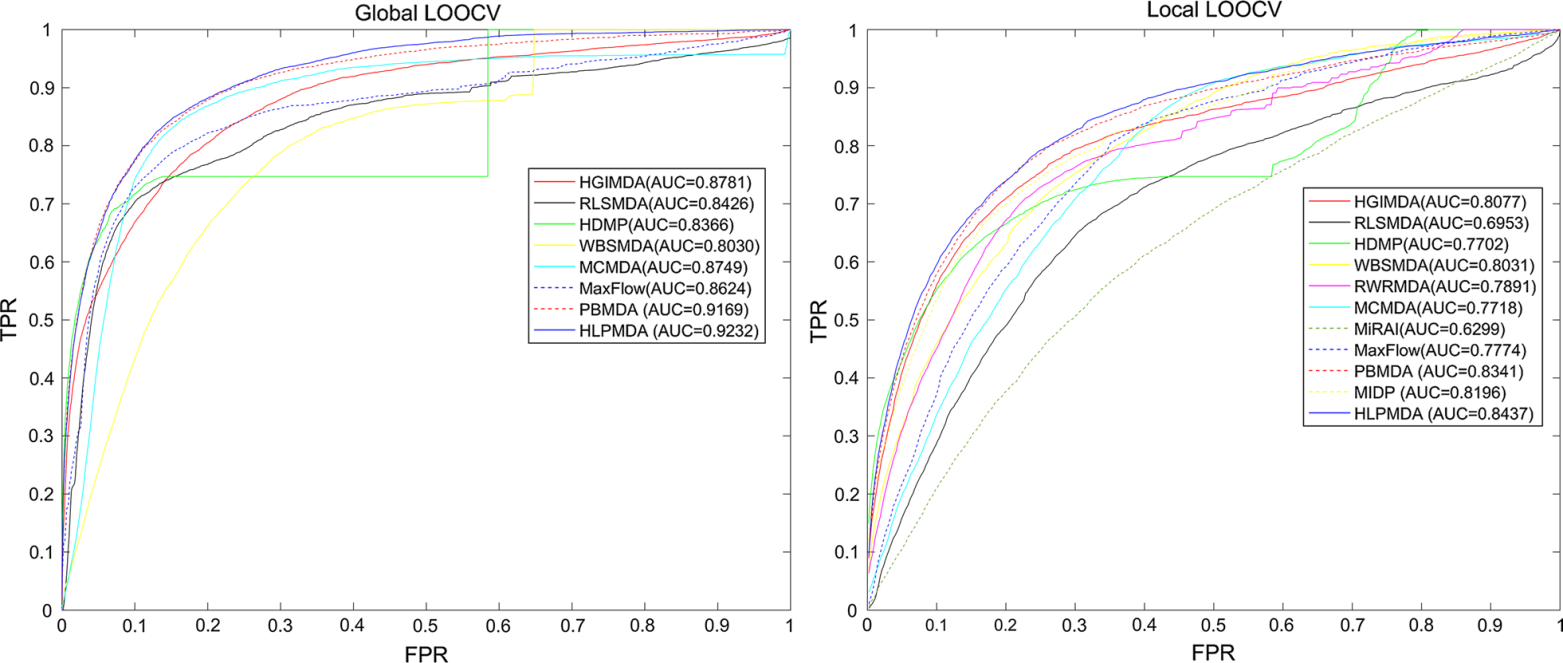
Predictive capability comparisons between HLPMDA and ten classical models of disease-miRNA association prediction (PBMDA, MCMDA, MaxFlow, HGIMDA, RLSMDA, HDMP, WBSMDA, MirAI, MIDP, and RWRMDA) in terms of ROC curve and AUC based on local and global LOOCV, respectively. As a result, HLPMDA achieved AUCs of 0.9232 and 0.8437 in the global and local LOOCV, significantly outperforming all the previous classical models

As illustrated in Fig. 3, HLPMDA achieved AUCs of 0.9232, 0.8437 and 0.9218 ± 0.0004 in the global LOOCV, local LOOCV and 5-fold CV, respectively, which shows a better predictive capability than other ten methods: PBMDA [52], MCMDA [50], MaxFlow [51], HGIMDA [49], RLSMDA [45], HDMP [38] WBSMDA [48], MirAI [47], MIDP [40] and RWRMDA [65]. (RWRMDA and MIDP are random walk-based method and this two method could be implemented only after determine the disease, so there are no global LOOCV results about them. MiRAI lacked the results of global LOOCV, either. Because during the caculation of MiRAI, the association scores for different diseases were not comparable.) Besides, MiRAI implemented on our data sets had a lower AUC (0.6299) than described in the origin literature [47], due to the data sparsity problem of collaborative filtering algorithm that MiRAI was based on.

### Case studies

To be specific, three malignant human diseases, esophageal neoplasms, breast neoplasms and Lymphoma were selected out to execute three kind of case studies (each kind of case studies investigate one disease).

In the first kind of case studies, data came from HMDD v2.0 and then the prediction results were checked up in miR2Disease [66] and dbDEMC database [67] (another two well-known miRNA-disease association databases). This kind of case studies is about esophageal neoplasms. Esophageal neoplasm is a common malignant tumor worldwide and it affects more males than females [68]. In terms of pathological characteristics, there are two main subtype of esophageal neoplasms: esophageal squamous cell carcinoma (ESCC) and esophageal adenocarcinoma (EAC) [68]. ESCC remains the main subtype of esophageal neoplasms [68]. Survival rate of esophageal neoplasms is improving but remains poor [69]. So more esophageal neoplasms related miRNAs may help detect, diagnose and treat esophageal neoplasms earlier. Until now, some miRNAs have been found associated with esophageal neoplasms. For example, after 24- and/or 72-h treatment of esophageal neoplasms by Chemotherapy, 13 miRNAs (miR-199a-5p, miR-302f, miR-320a, miR-342-3p, miR-425, miR-455-3p, miR-486-3p, miR-519c-5p, miR-548d-5p, miR-617, miR-758, miR-766, miR-1286) were deregulated [70]. By HLPMDA, the candidate miRNAs of esophageal neoplasms were ranked and then checked up by miR2Disease and dbDEMC. As a result, all of the top 10 and 47 out of the top 50 candidate miRNAs could be proved to be related with esophageal neoplasms (see Table 2). Besides, all candidate miRNAs were ranked by HLPMDA for all the diseases in HMDD v2.0 (see Additional file 1). We hope that these prediction results could help the corresponding experimental research in the future.

**Table 2:** HLPMDA was implemented to predict potential esophageal neoplasms-related miRNAs based on the known miRNA-disease association from HMDD v2.0 (left column: top 1–25; right column: top 26–50)

miRNA	Evidence	miRNA	Evidence
hsa-mir-125b	dbDEMC	hsa-mir-127	dbDEMC
hsa-mir-200b	dbDEMC	hsa-mir-429	dbDEMC
hsa-mir-17	dbDEMC	hsa-mir-181b	dbDEMC
hsa-mir-18a	dbDEMC	hsa-mir-181a	dbDEMC
hsa-mir-16	dbDEMC	hsa-mir-107	dbDEMC; miR2Disease
hsa-mir-221	dbDEMC	hsa-mir-24	dbDEMC
hsa-mir-1	dbDEMC	hsa-mir-182	dbDEMC
hsa-mir-222	dbDEMC	hsa-mir-30c	dbDEMC
hsa-let-7e	dbDEMC	hsa-mir-93	dbDEMC
hsa-let-7f	Unconfirmed	hsa-mir-18b	dbDEMC
hsa-mir-19b	dbDEMC	hsa-mir-199b	dbDEMC
hsa-mir-29a	dbDEMC	hsa-mir-133b	dbDEMC
hsa-let-7d	dbDEMC	hsa-mir-132	dbDEMC
hsa-let-7i	dbDEMC	hsa-mir-195	dbDEMC
hsa-mir-9	dbDEMC	hsa-mir-30a	dbDEMC
hsa-let-7 g	dbDEMC	hsa-mir-191	dbDEMC
hsa-mir-218	Unconfirmed	hsa-mir-15b	dbDEMC
hsa-mir-10b	dbDEMC	hsa-mir-302b	dbDEMC
hsa-mir-146b	dbDEMC	hsa-mir-224	dbDEMC
hsa-mir-142	dbDEMC	hsa-mir-194	dbDEMC; miR2Disease
hsa-mir-125a	dbDEMC	hsa-mir-20b	dbDEMC
hsa-mir-7	dbDEMC	hsa-mir-124	dbDEMC
hsa-mir-29b	dbDEMC	hsa-mir-302c	dbDEMC
hsa-mir-106a	dbDEMC	hsa-mir-122	Unconfirmed
hsa-mir-106b	dbDEMC	hsa-mir-335	dbDEMC

In the second kind of case studies, data also came from HMDD v2.0 but the investigated disease-related miRNAs were removed in order to evaluate the predictive capability for those diseases without any known associated miRNAs. Then the prediction results were checked up in HMDD v2.0, miR2Disease and dbDEMC database. This kind of case studies is about breast neoplasms. Breast neoplasms (Breast cancer) is the second leading cause of women cancer death in the US and the breast cancer death rates of black women remain higher than whites nationally [71]. Some miRNAs have been proved to correlate with Breast neoplasms and the corresponding treatment. For example, by decreasing TrkB and Bmi1 expression, miR-200c sensitizes breast cancer cells to doxorubicin treatment [72]. Furthermore, in human breast cancer cells miRNA-200 family alterations relates to mesenchymal and drug-resistant phenotypes [73]. By HLPMDA, the candidate miRNAs of Breast neoplasms were ranked and then checked up by HMDD v2.0, miR2Disease and dbDEMC. As a result, all of the top 10 and 49 out of the top 50 candidate miRNAs could be proved to be related with Breast neoplasms (see Table 3).

**Table 3:** HLPMDA was implemented to predict potential breast neoplasms-related miRNAs based on the known miRNA-disease association from HMDD v2.0 while the associations about breast neoplasms were removed and then the prediction results were checked up in HMDD v2.0, miR2Disease and dbDEMC database (left column: top 1–25; right column: top 26–50)

miRNA	Evidence	miRNA	Evidence
hsa-mir-21	HMDD dbDEMC miR2Disease	hsa-mir-205	HMDD; dbDEMC miR2Disease
hsa-mir-155	HMDD dbDEMC miR2Disease	hsa-mir-203	HMDD; dbDEMC miR2Disease
hsa-mir-125b	HMDD miR2Disease	hsa-mir-1	HMDD; dbDEMC
hsa-mir-145	HMDD; dbDEMC miR2Disease	hsa-mir-34c	HMDD; dbDEMC
hsa-mir-146a	HMDD dbDEMC miR2Disease	hsa-mir-19b	HMDD; dbDEMC
hsa-mir-221	HMDD dbDEMC miR2Disease	hsa-mir-375	HMDD; dbDEMC
hsa-mir-17	HMDD miR2Disease	hsa-mir-199a	HMDD; dbDEMC
hsa-mir-34a	HMDD dbDEMC	hsa-mir-100	HMDD; dbDEMC
hsa-let-7a	HMDD dbDEMC miR2Disease	hsa-mir-29a	HMDD; dbDEMC
hsa-mir-20a	HMDD miR2Disease	hsa-mir-210	HMDD; dbDEMC miR2Disease
hsa-mir-31	HMDD dbDEMC miR2Disease	hsa-mir-15a	HMDD; dbDEMC
hsa-mir-200c	HMDD dbDEMC miR2Disease	hsa-let-7c	HMDD; dbDEMC
hsa-mir-222	HMDD dbDEMC miR2Disease	hsa-mir-182	HMDD; dbDEMC miR2Disease
hsa-mir-126	HMDD dbDEMC miR2Disease	hsa-mir-34b	HMDD; dbDEMC
hsa-mir-18a	HMDD dbDEMC miR2Disease	hsa-mir-183	HMDD; dbDEMC
hsa-mir-92a	HMDD	hsa-mir-141	HMDD; dbDEMC miR2Disease
hsa-mir-200b	HMDD dbDEMC miR2Disease	hsa-mir-101	HMDD; dbDEMC miR2Disease
hsa-mir-16	HMDD dbDEMC	hsa-mir-146b	HMDD; dbDEMC miR2Disease
hsa-let-7b	HMDD dbDEMC	hsa-mir-181a	HMDD; dbDEMC miR2Disease
hsa-mir-142	Unconfirmed	hsa-let-7d	HMDD; dbDEMC miR2Disease
hsa-mir-9	HMDD dbDEMC miR2Disease	hsa-mir-10b	HMDD; dbDEMC miR2Disease
hsa-mir-200a	HMDD dbDEMC miR2Disease	hsa-let-7e	HMDD; dbDEMC
hsa-mir-223	HMDD dbDEMC	hsa-mir-29c	HMDD; dbDEMC miR2Disease
hsa-mir-19a	HMDD dbDEMC	hsa-mir-218	HMDD; dbDEMC
hsa-mir-143	HMDD dbDEMC miR2Disease	hsa-let-7 g	HMDD; dbDEMC

In the third kind of case studies, data came from HMDD v1.0 and then the prediction results were checked up in HMDD v2.0, miR2Disease and dbDEMC database, just for the sake of examining the robustness of HLPMDA on the different dataset. This kind of case studies is about Lymphoma originating in the lymphatic hematopoietic system, which accounts for more than one-fifth of all cancer cases [71]. According to the tumor cells, there are two categories of lymphoma: Hodgkin lymphomas (HL) and the non-Hodgkin lymphomas (NHL) [74, 75]. It is very hard for HL to be detected at early stages [74, 75]. Some miRNAs were found associated with lymphoma. For instance, there are different expressions of miR-150 between lymphoma and small lymphocytic leukemia [76], and specifically, miR-150 is a tumor suppressor in malignant lymphoma [77]. Besides, EBV-positive Burkitt lymphoma differentiation can be induced by re-expression of miR-150 targeting c-Myb [78]. By HLPMDA, the candidate miRNAs of lymphoma were ranked and then checked up by HMDD v2.0, miR2Disease and dbDEMC. As a result, 9 of the top 10 and 46 out of the top 50 candidate miRNAs could be proved to be related with lymphoma (see Table 4).

**Table 4:** HLPMDA was implemented to predict potential lymphoma-related miRNAs based on the known miRNA-disease association from HMDD v1.0 and then the prediction results were checked up in HMDD v2.0, miR2Disease and dbDEMC database (left column: top 1–25; right column: top 26–50)

miRNA	Evidence	miRNA	Evidence
hsa-mir-21	HMDD; dbDEMC	hsa-mir-127	dbDEMC
hsa-mir-155	HMDD; dbDEMC	hsa-mir-141	dbDEMC
hsa-mir-221	dbDEMC	hsa-mir-199a	dbDEMC
hsa-let-7a	dbDEMC	hsa-mir-29c	HMDD; dbDEMC
hsa-mir-146a	HMDD; dbDEMC	hsa-mir-24	HMDD; dbDEMC
hsa-mir-125b	Unconfirmed	hsa-mir-146b	Unconfirmed
hsa-mir-222	dbDEMC	hsa-mir-200a	HMDD; dbDEMC
hsa-mir-34a	dbDEMC	hsa-mir-143	dbDEMC
hsa-let-7b	dbDEMC	hsa-mir-181a	HMDD; dbDEMC
hsa-let-7e	dbDEMC	hsa-mir-25	dbDEMC
hsa-mir-223	dbDEMC	hsa-mir-150	HMDD; dbDEMC
hsa-let-7d	dbDEMC	hsa-mir-106a	dbDEMC
hsa-mir-29b	dbDEMC	hsa-mir-125a	HMDD; dbDEMC
hsa-mir-29a	dbDEMC	hsa-mir-373	dbDEMC
hsa-mir-145	dbDEMC	hsa-mir-101	HMDD; dbDEMC
hsa-mir-9	dbDEMC	hsa-mir-451	dbDEMC
hsa-mir-200b	HMDD; dbDEMC	hsa-mir-93	dbDEMC
hsa-let-7i	dbDEMC	hsa-mir-214	dbDEMC
hsa-let-7f	dbDEMC	hsa-mir-34c	Unconfirmed
hsa-let-7c	dbDEMC	hsa-mir-34b	dbDEMC
hsa-let-7 g	dbDEMC	hsa-mir-191	dbDEMC
hsa-mir-126	HMDD; dbDEMC	hsa-mir-339	dbDEMC
hsa-mir-181b	dbDEMC	hsa-mir-194	dbDEMC
hsa-mir-106b	dbDEMC	hsa-mir-132	dbDEMC
hsa-mir-1	dbDEMC	hsa-mir-429	Unconfirmed

## Discussion

The reliability and availability of HLPMDA lied in the following several aspects. Firstly, HMDD as well as other biological datasets provided a solid foundation for the subsequent prediction steps. Secondly, the introduction of lncRNA data and the application of bipartite network projection help profile the relationship between one miRNA and another miRNA, between one disease and another disease. There is a widely accepted view that more data may help produce a better output. Adding the corresponding lncRNA data brings more information to the problem of latent miRNA-disease association prediction. It is a fresh perspective and it was proved to be an advantageous improvement by the performance of HLPMDA. Bipartite network projection also dug out more implicit message that made the prediction more accurate. In addition, the heterogeneous label propagation is a useful algorithm based on the local and global feature in the constructed network, with no need of negative examples. In recent years, the network approach has been relatively widely adopted in some fields of bioinformatics [79–81]. The major cause is that similarity, links, associations, interactions and relationships among the research targets (like miRNA, diseases and so on) in the network approach become easier to be represented, calculated, analyzed and tested by some math tools, together with some descriptive expressions transformed into quantitative representations. As a result, it indeed helps improve the effectiveness of the prediction. Finally, according to NanoString’s Hallmarks of Cancer Panel collection (https://www.nanostring.com/), it is proved that a part of the miRNAs’ targets is related to cancer hallmarks [82, 83], which were found to be associated with the corresponding genes. So our work may be helpful for the further research about cancer hallmarks, genes and miRNA.

However, HLPMDA is undeniably limited by following factors which are also the room to improve HLPMDA. First, the data about miRNA and disease is not ample enough. For instance, the known miRNA-disease associations have a large degree of sparsity (labeled miRNA-disease associations only accounts for 2.86% of 189,585 miRNA-disease pairs). It is believed that more data could promote the performance of the computational model. Therefore, with more information about miRNA, disease and some other objects (like genes, drugs, targets and so on) related to one or both of them put to use [84], predictive power of HLPMDA would be stronger. Second, it may be unfair for different miRNAs or diseases because the known information about every item is not relatively equivalent. Therefore, HLPMDA may cause advantageous bias to miRNAs or diseases which have more known association (or interaction) records. Last but not the least, the parameters in HLPMDA were set according to the previous similar studies and our experience. We have not thought a lot of the parameters but there may exist better parameters which could bring about more accurate prediction results.

Data collection, database construction, data analysis, mining and testing about miRNA-disease associations has become an important field in bioinformatics. As we all know, there are strong connections in many fields of biology. The research of miRNA-disease association relates to protein–protein interaction, miRNA-target interaction, miRNA–lncRNA interaction, drug, environmental factor, etc. In the future, we believe that this field need to obtain more data and to be integrated with other research areas for the sake of producing predictive synergy with more integrated data.

## Conclusion

It is valuable to seek the underlying miRNA-disease associations. In this paper, on the grounds that functionally similar miRNAs were likely to correlate with similar diseases and vice versa, heterogeneous label propagation for MiRNA-disease association prediction (HLPMDA) was proposed. AUCs of HLPMDA are 0.9232 (global LOOCV), 0.8437 (local LOOCV) and 0.9218 ± 0.0004 (5-fold CV). In three case studies, the accurate rates were all higher than 85%. Furthermore, three kinds of case studies were implemented for further evaluations. As a result, 47 (esophageal neoplasms), 49 (breast neoplasms) and 46 (lymphoma) of top 50 candidate miRNAs were proved by experiment reports. All the results sufficiently showed the reliability of HLPMDA in predicting possible disease-miRNA associations. HLPMDA will be a valuable computational tool for miRNA-disease association prediction and miRNA biomarker identification for human disease.

## Additional file

**Additional file 1.** All candidate miRNAs were ranked by HLPMDA for all the diseases in HMDD v2.0. Prediction results could be obtained publicly for further research and experimental validation.

## Abbreviations

MiRNA: microRNA
LncRNA: long non-coding RNA
LOOCV: leave-one-out cross validation
5-fold CV: 5-fold cross validation
ROC: receiver-operating characteristics curve
AUC: the area under ROC curve
